# 97. Hemophagocytic Lymphohistiocytosis in Disseminated Histoplasmosis: an Overlooked Diagnosis

**DOI:** 10.1093/ofid/ofae631.034

**Published:** 2025-01-29

**Authors:** Burton Mandrell, Tatsiana Savenka, Michael Saccente

**Affiliations:** University of Arkansas for the Medical Sciences, Little Rock, AR; University of Arkansas for the Medical Sciences, Little Rock, AR; University of Arkansas for Medical Sciences, Little Rock, Arkansas

## Abstract

**Background:**

Hemophagocytic lymphohistiocytosis (HLH) is a life-threatening syndrome involving pathologic excitation of the immune system. Disseminated histoplasmosis (DH) is a known trigger of HLH. However, the prevalence of HLH in DH is not known. Limited data exist on risk factors and outcomes. The goals of this study are to determine the prevalence of HLH among participants with DH, identify risk factors for HLH in this population, and describe the treatment and outcomes of people with DH and HLH.

**Figure:**
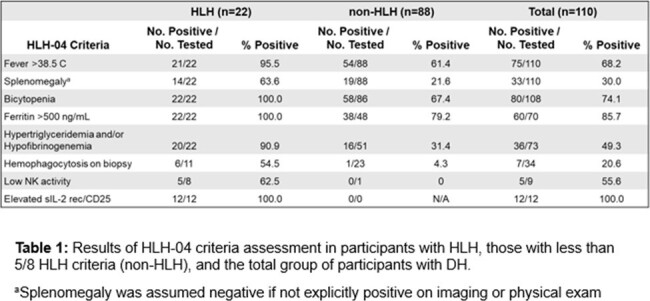

**Methods:**

We retrospectively identified 110 cases of DH at our institution from 2014 to 2022. The diagnosis of DH required culture of *Histoplasma capsulatum* from a non-pulmonary site, or non-pulmonary tissue histopathology with yeast identifiable as *Histoplasma*, or a combination of clinical abnormalities consistent with DH plus detection of *Histoplasma* antigen in urine or blood (MiraVista Diagnostics). Established HLH-04 criteria defined HLH. Continuous variables are presented as medians with interquartile ranges. Categorical variables are compared with Fisher’s exact test or Mann-Whitney U test. We used odds ratios to identify potential predictors of HLH. P values < 0.05 were considered significant.

**Figure:**
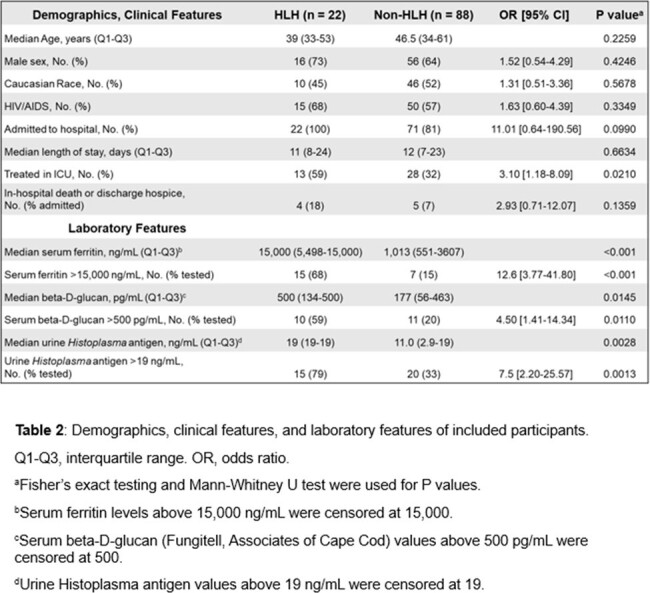

**Results:**

Among 110 participants with DH, 22 met criteria for HLH (Table 1). In the subset who were hospitalized, 23.7% (22/93) had HLH. Compared to participants without HLH, the HLH cohort was more likely to have serum ferritin above the limit of quantification (LOQ) ( > 15,000 ng/ml), urine *Histoplasma* antigen above the LOQ ( > 19 ng/mL), serum beta-D-glucan (BDG) above the LOQ ( > 500 pg/mL), and more likely to be treated in the ICU (Table 2). There was no significant difference in HIV/AIDS status, race, sex, or in-hospital death/transition to hospice. All 22 participants identified with HLH received liposomal amphotericin B. 2 participants with HLH received etoposide, and 1 received IVIG.

**Conclusion:**

Nearly a quarter of participants with DH admitted to our hospital had HLH. This is likely an underestimate because most were not assessed for every HLH criterion. Serum ferritin > 15,000 ng/ml, urine *Histoplasma* antigen above the LOQ, and serum BDG above the LOQ should prompt investigation for HLH. Further studies are needed to assess optimal treatment strategies in this population.

**Disclosures:**

**All Authors**: No reported disclosures

